# Screening for Prediabetes Using Machine Learning Models

**DOI:** 10.1155/2014/618976

**Published:** 2014-07-16

**Authors:** Soo Beom Choi, Won Jae Kim, Tae Keun Yoo, Jee Soo Park, Jai Won Chung, Yong-ho Lee, Eun Seok Kang, Deok Won Kim

**Affiliations:** ^1^Department of Medical Engineering, Yonsei University College of Medicine, 50-1 Yonsei-ro, Seodaemun-gu, Seoul 120-752, Republic of Korea; ^2^Brain Korea 21 PLUS Project for Medical Science, Yonsei University, Republic of Korea; ^3^Department of Medicine, Yonsei University College of Medicine, Republic of Korea; ^4^Graduate Program in Biomedical Engineering, Yonsei University, Seoul, Republic of Korea; ^5^Department of Internal Medicine, Yonsei University Health System, Republic of Korea

## Abstract

The global prevalence of diabetes is rapidly increasing. Studies support the necessity of screening and interventions for prediabetes, which could result in serious complications and diabetes. This study aimed at developing an intelligence-based screening model for prediabetes. Data from the Korean National Health and Nutrition Examination Survey (KNHANES) were used, excluding subjects with diabetes. The KNHANES 2010 data (*n* = 4685) were used for training and internal validation, while data from KNHANES 2011 (*n* = 4566) were used for external validation. We developed two models to screen for prediabetes using an artificial neural network (ANN) and support vector machine (SVM) and performed a systematic evaluation of the models using internal and external validation. We compared the performance of our models with that of a screening score model based on logistic regression analysis for prediabetes that had been developed previously. The SVM model showed the areas under the curve of 0.731 in the external datasets, which is higher than those of the ANN model (0.729) and the screening score model (0.712), respectively. The prescreening methods developed in this study performed better than the screening score model that had been developed previously and may be more effective method for prediabetes screening.

## 1. Introduction

The prevalence of type 2 diabetes is dramatically increasing, resulting in a global public health issue [1]. The prevalence of diabetes was estimated at 285 million or 6.4% of adults in the world in 2010 [2], and this prevalence is expected to rise to 552 million by 2030 [3]. The increasing rates of obesity are expected to result in a faster increase in the prevalence of type 2 diabetes in the future [4]. However, owing to the absence of symptoms and/or disease-related knowledge, diabetes often goes undetected, and approximately one-third of people with diabetes are not aware of their status [5–7]. Therefore, development of a simple accurate screening method is needed. Historically, the majority of the clinical screening methods consisted of surveys developed using logistic regression analyses to predict diabetes [8–13].

Prediabetes was first recognized as an intermediate diagnosis and indication of a relatively high risk for the future development of diabetes by the Expert Committee on Diagnosis and Classification of Diabetes Mellitus in 1997 [14], and it has been reported that approximately 5–10% of patients with untreated prediabetes subsequently develop diabetes [15, 16]. This is significant considering that prediabetes based on impaired fasting glucose (IFG) was estimated to affect 4.9 million people, accounting for 17.4% of Korean adults in 2005 [6], with a further 35% of adults in the US with prediabetes in 2008 [17]. The definition of prediabetes includes a fasting plasma glucose (FPG) level in the range of 100–125 mg/dL (5.6–6.9 mmol/L), impaired glucose tolerance (IGT) (oral glucose tolerance test (OGTT) 2 h measurement in the range of 140–199 mg/dL (7.8–11.0 mmol/L)), or HbA1c level in the range of 5.7–6.4% (39–46 mmol/mol). Similar to diabetes, the risk of microvascular complications is increased with prediabetes [18], and the risk for cardiovascular disease and total mortality is almost twice as high in individuals with prediabetes [19, 20]. Early diagnosis and intervention for prediabetes could prevent these complications, prevent delay, or prevent the transition to diabetes [21] and be cost-effective [22].

Machine learning is an area of artificial intelligence research, which uses statistical methods for data classification. Several machine learning techniques have been applied in clinical settings to predict disease and have shown higher accuracy for diagnosis than classical methods [23]. Support vector machines (SVM) and artificial neural networks (ANN) have been widely used approaches in machine learning. They are the most frequently used supervised learning methods for analyzing complex medical data [24].

In this study, we aimed to develop and validate models to predict prediabetes using artificial neural network (ANN) and support vector machine (SVM) methods, which could be effective as simple and accurate screening tools. The model performance was compared to that of the screening score model that we modified for prediabetes based on the screening score for diabetes by Lee et al. [8], with respect to accuracy and area under the curve (AUC) of the receiver operating characteristic (ROC).

## 2. Materials and Methods

### 2.1. Data Source and Subjects

**Figure 1:**
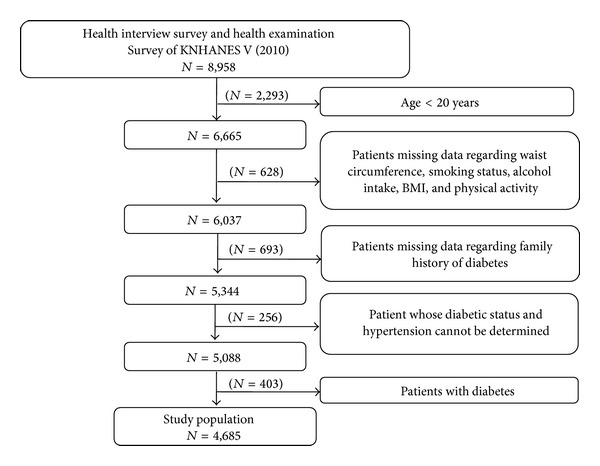
Flow chart of excluding subjects for the KNHANES 2010.

Data from the Korean National Health and Nutrition Examination Survey (KNHANES) 2010 [25] and 2011 [26] were used to develop and validate, respectively, the ANN and SVM models for prediabetes. The KNHANES is a cross-sectional survey that includes approximately 800 questions; it is conducted by the Division of Chronic Disease Surveillance, Korea Centers for Disease Control and Prevention. The survey represents the entire nation using rolling sampling survey method. The following exclusion criteria applied to the subjects in both datasets: <20 years of age, missing data for waist circumference, smoking status, alcohol intake, body mass index (BMI), physical activity, family history of diabetes, undetermined diabetic status or hypertension status, and diagnosed diabetes or undiagnosed diabetes. Diagnosed and undiagnosed patients were excluded in order to focusing on predicting prediabetes. Undiagnosed diabetes was defined as a FPG ≥ 126 mg/dL without diagnosis by clinician. Of the 8,958 subjects in the KNHANES 2010, 4,685 were included as shown in Figure 1. Of the 8,518 subjects in the KNHANES 2011, 4,566 subjects were included using the same flow chart as Figure 1. The subjects of the KNHANES 2010 and 2011 data sets were not overlapped.

**Figure 2:**
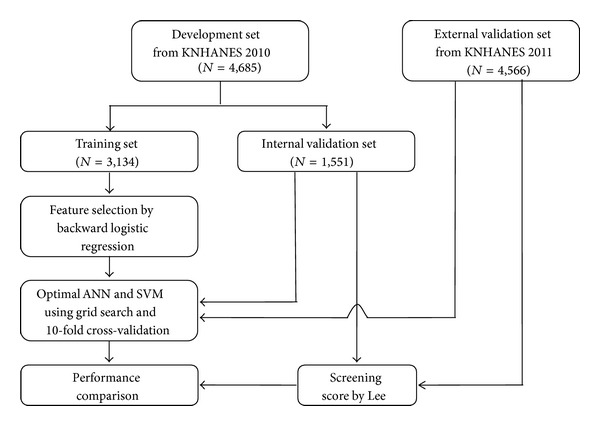
Chart depicting the flow of data from the Korean National Health and Nutrition Examination Survey (KNHANES) 2010 and 2011 to develop and validate a prediabetes model. KNHANES: Korean National Health and Nutrition Examination Survey; ANN: artificial neural network; SVM: support vector machine.

Figure 2 illustrates the study flow. The development dataset from KNHANES 2010 was randomly divided into training and internal validation sets using a 2 : 1 ratio. The training set (*n* = 3, 134) was used to construct the ANN and SVM models. The internal validation set (*n* = 1, 551) was used to assess the ability to predict prediabetes. Additionally, data from KNHANES 2011 were used as an external validation set (*n* = 4, 566). All individuals in the surveys participated voluntarily and informed consent was obtained from all participants. The survey protocol was approved by the Institutional Review Board of the Korean Centers for Disease Control and Prevention.

### 2.2. Risk Factors

We adopted the most frequently used nine variables from previous studies regarding diabetes prediction models: age, gender, family history of diabetes, hypertension, alcohol intake, BMI, smoking status, waist circumference, and physical activity [8–13]. FPG was determined using glucose levels that were collected following ≥ 8 hours of fasting. We considered only FPG although there are three methods to diagnose prediabetes. In KNHANES 2010, FPG was obtained from every subject, but OGTT was not tested, and HbA1c was tested only for subjects with diabetes. A family history of diabetes was limited to parents and siblings. Hypertension was defined as systolic blood pressure (SBP) > 140 mmHg, diastolic blood pressure (DBP) > 90 mmHg, or use of medication for blood pressure control [8]. Alcohol intake was calculated using 2 questions: (1) alcohol consumption frequency during the previous 12 months and (2) average number of drinks on those days. The amount of alcohol was calculated based on the number of glasses, regardless of the kind of beverage, assuming that the amount of alcohol was approximately the same in each glass (approximately 8 g alcohol per glass). Smoking status was divided into “currently smoking regularly” and “others,” with the latter group including subjects who had never smoked or had quit smoking. The subjects who answered more than “moderate” to the question “how intense is your everyday activity?” were considered as physically active.

### 2.3. Artificial Neural Network

ANN is an artificial intelligence technology, inspired by the architecture of biological neurons such as that in the human brain [27]. The technology is specialized for classification, and it is mostly used to identify underlying patterns for risk factors in medicine. When trained properly, neural networks are known to have more accurate predictive abilities than conventional methods such as logistic regression. There have been a number of recent advances in ANN methodology that enable automatic detection of an optimal predictive model [28, 29]. Unlike logistic regression, ANNs are able to detect complex nonlinear relationships between multiple predictors and diseases, which make them useful in support systems for medical decisions [30, 31].

The ANN models were constructed using NeuroSolution version 6.0 (NeuroDimension, Gainesville, FL), which is professional software that simplifies the construction of ANN [32]. This software allows simultaneous testing of various types of neural networks, including generalized regression neural network, multilayer perceptron, probabilistic neural network, radial basis neural network, feedforward neural network, and support vector machine. To avoid overfitting, the prediction models were internally validated using cross-validation. Performance of the prediction models was monitored during training and cross-validation to obtain optimal algorithm and parameters, such as learning rate, momentum, and number of hidden nodes. The ANN was trained with 7 predictors including age, gender, waist circumference, BMI, family history of diabetes, hypertension, and alcohol intake, which were selected using backward logistic regression. The model chosen for prediabetes prediction was a multilayer perceptron model with 1 hidden layer, batch training, and momentum learning (MLP-1-BM) of backpropagation feedforward algorithm, which demonstrated the best performance as a desired ANN.

### 2.4. Support Vector Machine

SVM maps data to a higher dimensional space through a kernel function that linearly separates data patterns. The data are divided into two groups by the training data referred to as a support vector. SVM models are determined by choosing the maximum-margin hyperplane with the nearest support vector of the two groups [33]. SVM improves the accuracy of a model through the optimization of separating space using the kernel function, but one of the disadvantages of SVM is that it requires many trials to construct an optimal SVM model in comparison with other machine learning techniques [34].

The same seven risk factors as those in the ANN model were employed for the SVM. To obtain the optimal model, we adopted a grid search in which a range of parameter values (penalty parameter [*C*] of 0.01, 0.1, 1, 10, and 100 and scaling factor [σ] of 0.001, 0.01, 0.1, 1, 10, and 100) was tested using the 10-fold cross-validation strategy. The optimal parameter values with a *C* of 10 and σ of 10 for SVM using the Gaussian kernel function were obtained. The SVM models were constructed using MATLAB Version 2012a (Mathworks Inc., Natick, MA).

### 2.5. Screening Score of Our Models for Prediabetes

The models constructed by ANN and SVM were compared with a previously developed screening survey to illustrate performance of our models and the possibility of their use in real situations. For this purpose, we used a screening score model for the Korean population constructed by Lee et al. [8]; we felt this was appropriate because both studies constructed models for the Korean population. Lee et al. used data from KNHANES 2001 and 2005 for training and data from KNHANES 2007 and 2008 for external validation. In addition, the screening score model by Lee et al. used very similar risk factors to ours, with the exception of current smoking status. Those 6 variables independently associated with undiagnosed diabetes were chosen for their model: age, family history of diabetes, hypertension, waist circumference, smoking, and alcohol intake.

The risk score was assigned according to the odds ratio for each risk factor in the logistic regression model defined by Lee et al. [8]. Within the total score range of 0–11 points, a cut-off score of ≥5 points was selected to indicate an individual at high risk for undiagnosed diabetes; this cut-off resulted in the highest value for the Youden index. The 6 risk factors jointly yielded an AUC of 0.730 for both the internal and external validation sets [8]. To compare with our models for prediabetes, we constructed a new screening score model for prediabetes by adjusting the cut-off point value based on our definition of prediabetes (100 mg/dL ≤ FPG < 126 mg/dL), given that the screening score for diabetes used by Lee et al. was based on FPG ≥ 126 mg/dL [8]. The screening score for prediabetes was designed with the same risk score model of the 6 risk factors using our training set for prediabetes (KNHANES 2010) and the Youden index; as a result, a cut-off score of ≥5 points was identified to indicate an individual with prediabetes.

### 2.6. Statistical Analyses

The weighted characteristics of the data from the KNHANES 2010 to represent the entire normal and prediabetes people in Korea are summarized by descriptive statistics in Table 1. For comparison of the factors between normal and prediabetes, the continuous and categorical characteristics were tested using *t*-test and chi-square test, respectively.

To obtain the optimal variables for the prediction model, backward logistic regression was performed with the training set. Each step of the backward regression excluded the variables without a statistically meaningful correlation with the outcome, prediabetes. Three steps of backward regression were executed, and the selected 7 variables were age, BMI, hypertension, gender, alcohol intake, waist circumference, and family history. The Hosmer-Lemeshow test resulted in a *P* value of 0.132, indicating that the chosen variables were well fitted.

ROC curve analysis is the most commonly used method in clinical analysis to establish an optimal cut-off point [35]. Therefore, we generated ROC curves and the selected cut-off points that maximized the Youden index [36] to compare the performance of our optimal machine learning models with that of the screening score model for prediabetes based on the screening score by Lee et al. [8], using our internal and external validation sets. Following the ROC analysis, the AUC, accuracy, sensitivity, and specificity of our models and screening score model for prediabetes were calculated. The classification accuracy measured the proportion of cases correctly classified. Sensitivity measured the fraction of positive cases that were classified as positive. Specificity measured the fraction of negative cases that were classified as negative. We used SPSS 20.0 (IBM Corp, Armonk, NY) for statistical analysis and MedCalc 12.4 (MedCalc Inc., Mariakerke, Belgium) for ROC analysis. Statistical significance was set at *P* < 0.05.

## 3. Results

The weighted characteristics of the KNHANES 2010 data are summarized in Table 1. The factors that were significantly related to prediabetes were age, gender, family history of diabetes, alcohol intake, BMI, waist circumference, FPG, systolic and diastolic blood pressures, and hypertension.

When the prediction performance of the ANN model using 10-fold cross-validation was assessed for the training set, the final model showed an AUC of 0.706 and an accuracy of 65.6% for prediabetes. Cross-validation of the optimal SVM parameters with the training set resulted in an AUC of 0.742 and accuracy of 69.9%. These results are not included in Table 2. The similar performance observed between the training and validation sets in Table 2 indicates that the trained models were not overfitting.

**Figure 3:**
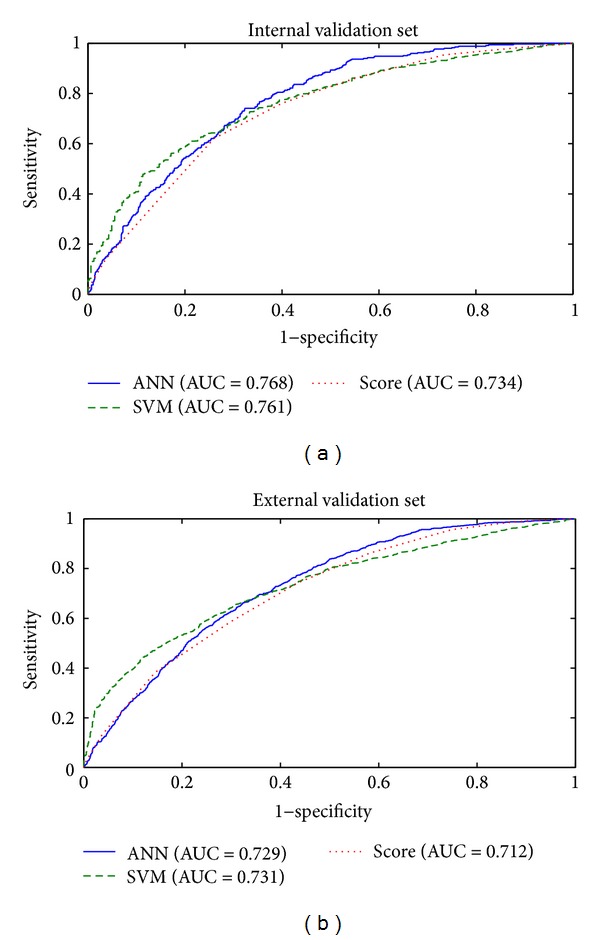
Receiver operating characteristic curves (ROC) of artificial neural network (ANN), support vector machine (SVM), and screening score in predicting prediabetes for internal validation set (a) and external validation set (b).

With both the internal and the external validation sets, our ANN and SVM models showed better performance than the existing screening score model using logistic regression, especially in terms of AUC, which is known as a better measure than accuracy in evaluating learning algorithms [37] (Table 2). In the external validation set, the accuracy of the SVM model was 5.4% and 6.2% higher than that of the ANN and screening score models, respectively. The ROC curves of the ANN, SVM, and screening score models are depicted for the internal and external validation sets in Figure 3.

Table 3 shows the performance obtained by applying the screening score model by Lee et al. [8] to the data from KNHANES 2010 and 2011 for predicting prediabetes and undiagnosed diabetes. For all performance parameters (AUC, accuracy, sensitivity, and specificity) in both datasets, the ability to predict prediabetes was inferior to that for diabetes. In particular, the AUC and accuracy for prediabetes in the external validation set were lower than those for undiagnosed diabetes by 0.039 and 4.7%, respectively. AUC and accuracy of the SVM model for external validation are higher than those of the screen score model for prediabetes by 0.019 and 6.2%, respectively.

## 4. Discussion

The results of the present study indicate that the ANN and SVM models that we developed to predict prediabetes, defined as IFG, performed better than the existing clinical screening score model, as indicated by the AUC and accuracy measures (Table 2). Although logistic regression analysis and ANN share common roots in statistical pattern recognition, the latter is a generalization of the former [38], which might explain why our ANN model performed better than the screening score model, which was based on logistic regression analysis. The SVM model performed particularly well due to the ability of SVM to efficiently find a unique optimal solution, incorporate multiple types of data with a degree of flexibility, and model nonlinear patterns [39]. We also investigated SVM models with different numbers of risk factors to find optimal parameters. The best performance of SVM model with six risk factors including age, body mass index, hypertension, gender, daily alcohol intake, and waist circumference was an AUC of 0.743 and accuracy of 70.2% in training set, which was almost the same as the performance of SVM model with seven risk factors in our paper, resulting in an AUC of 0.742 and accuracy of 69.9%.

Meng et al. [40] compared the performance of logistic regression, ANNs, and decision tree models for predicting diabetes or prediabetes using common risk factors in China population. In Meng et al. study, the ANNs model was the poorest of the three models, with 73.23% accuracy. This result is consistent with ours that the performance of ANN model was lower than SVM model. However, the performance of ANN can be case-by-case depending on characteristics of data or developers.

Although similar statistical analyses were conducted (i.e., backward regression models), there were slight differences in the variables included in the present study and those in the study by Lee et al. [8]. Lee et al. included current smoking status as a risk factor in the training set based on the data from KNHANES 2001 and 2005; however, current smoking status was not included in our training set using data from KNHANES 2010. This may have resulted from lifestyle changes in the Korean population between those years, including a decline in the overall smoking rate and stronger antismoking laws [41]. Although several screening score models have been developed and used clinically, our prediction model is unique in several ways.

First, owing to the similarity between our machine learning models and the existing screening score models, we were able to compare the performance of our machine learning models with the existing models. Second, to the best of our knowledge, there are few studies investigating prediabetes; instead, the majority of the other models have been developed to predict undiagnosed diabetes. However, prediabetes is increasingly becoming a significant public health issue. Using our model to screen patients for prediabetes would enable interventions at an earlier stage, which would be easier to implement and more successful than interventions implemented following diabetes screening.

Prediabetes was more difficult to predict than diabetes using any of the parameters across all of the models, which is not unexpected. AUC and accuracy of the SVM model for external validation are higher than those of the screen score model for prediabetes by 0.019 and 6.2%, respectively (Table 2). Therefore, we demonstrated that the machine learning methods could help to overcome the difficulty in predicting prediabetes.

This study has certain limitations. First, FPG level was the only measurement that we used to define prediabetes and diabetes; OGTT and HbA1c were not taken into consideration. Data were lacking for these measurements; however, the use of FPG level was consistent with the model developed by Lee et al. [8], with which we compared our models.

Second, the screening score model for diabetes developed by Lee et al. [8] did not correspond perfectly with our model for prediabetes. For a more precise comparison in future studies, a screening score model for prediabetes should be constructed using the new regression equation with different risk factors for prediabetes. In spite of this limitation, the suggested model with the new cut-off point is considered a good model for predicting prediabetes with AUCs of 0.734 and 0.712 in the internal and external validation sets, respectively.

Last, the new models that we developed are limited in terms of convenience and potential widespread use. Although the screening score model is not the most effective one for disease prediction, it is simple and accessible. However, machine learning models could also become more accessible through the use of calculator software, particularly with the widespread use of devices such as computers, smart phones, and tablet PCs. Future studies could develop a calculator in which the values are entered via a website or application and the results are immediately delivered to the end user. The decision tree method is also warranted for easy interpreting tree-like plot in the future.

## 5. Conclusion

Our study constructed a reasonably good model to predict prediabetes in the Korean population. By applying similar methods in other countries, researchers could develop country-specific machine learning models for nationwide use. The creation of a user-friendly calculator program would enable access to screening by the general population, in addition to medical professionals. This widespread use could result in early diagnosis and treatment for people with prediabetes and diabetes, helping to relieve the public health diabetes burden and reducing the number of people who remain undiagnosed.

## Figures and Tables

**Table 1:** The weighted characteristics of the data from the Korean National Health and Nutrition Examination Survey (KNHANES) 2010.

	Normal (*n* = 3,681)	Prediabetes (*n* = 1,004)	P*
Age (years)	41.9 ± 0.5 [41.0–42.8]	52.5 ± 0.6 [51.3–53.7]	<0.001
Gender (% men)	46.9 (0.9) [45.2–48.7]	58.8 (1.9) [55.0–62.5]	<0.001
Family history of diabetes (%)	18.3 (0.9) [16.6–20.2]	22.9 (1.7) [19.8–26.4]	0.007
Current smoker (%)	27.5 (1.0) [25.5–29.6]	26.9 (1.9) [23.4–30.8]	0.799
Alcohol intake (drinks/day)	0.8 ± 0.0 [0.7–0.9]	1.0 ± 0.1 [0.9–1.2]	<0.001
Physically active (%)	50.6 ± 1.1 [48.4–52.9]	52.1 ± 2.1 [48.0–56.3]	0.535
BMI (kg/m^2^)	23.2 ± 0.1 [23.1–23.3]	25.1 ± 0.1 [24.8–25.3]	<0.001
Waist circumference (cm)	79.1 ± 0.2 [78.7–79.6]	85.8 ± 0.4 [85.1–86.6]	<0.001
FPG (mg/dL)	89.0 ± 0.1 [88.7–89.3]	107.4 ± 0.3 [106.9–108.0]	<0.001
Systolic blood pressure (mmHg)	116.8 ± 0.4 [116.0–117.5]	127.7 ± 0.7 [126.4–129.1]	<0.001
Diastolic blood pressure (mmHg)	76.4 ± 0.3 [75.8–77.0]	81.5 ± 0.5 [80.6–82.4]	<0.001
Hypertension (%)	16.4 (0.8) [14.9–18.0]	41.1 (2.2) [36.8–45.5]	<0.001

BMI: body mass index; FPG: fasting plasma glucose.

Table values are given as mean ± standard error or % (standard error) [95% confidence interval] unless otherwise indicated. P* were obtained by *t*-test or chi-square test.

Impaired fasting glucose was considered with values ≥ 100 mg/dL and <126 mg/dL.

**Table 2:** Performance of the ANN, SVM, and screening score (Lee et al. [8]) models using the internal and external validation sets for predicting prediabetes.

		AUC	Accuracy (%)	Sensitivity (%)	Specificity (%)
Internal validation set (*n* = 1,551)	ANN∗	0.768	69.0	74.1	67.5
	SVM^†^	0.761	64.9	78.9	61.2
	Screening score^‡^	0.734	63.4	76.1	60.0
External validation set (*n* = 4,566)	ANN∗	0.729	60.7	77.2	56.7
	SVM^†^	0.731	66.1	69.4	65.3
	Screening score^‡^	0.712	59.9	74.3	56.4

AUC: area under the curve; ANN: artificial neural network; SVM: support vector machine.

The internal validation set was comprised of data from the Korean National Health and Nutrition Examination Survey (KNHANES) 2010, and the external validation set included data from KNHANES 2011. ∗The chosen model was a multilayer perceptron model with 1 hidden layer, batch training, and momentum learning (MLP-1-B-M) of backpropagation feedforward algorithm. ^†^The optimal model was found using Gaussian kernel function with a penalty parameter (C) of 10 and scaling factor (σ) of 10. ^‡^The performance was calculated by applying the screening score model for prediabetes based on that of Lee et al. [8] to the data from KNHANES 2010 and 2011.

**Table 3:** Performance of the screening score model (Lee et al. [8]) in predicting prediabetes and undiagnosed diabetes using the data from the Korean National Health and Nutrition Examination Survey (KNHANES) 2010 and 2011.

		AUC	Accuracy (%)	Sensitivity (%)	Specificity (%)
Prediabetes	KNHANES 2010∗(internal validation)	0.734	63.4	76.1	60.0
	KNHANES 2011∗(external validation)	0.712	59.9	74.3	56.4
Undiagnosed diabetes	KNHANES 2010^†^(internal validation)	0.772	66.6	76.5	66.4
	KNHANES 2011^†^(external validation)	0.751	64.6	74.4	64.3

AUC: area under the curve; KNHANES: Korean National Health and Nutrition Examination Survey.

Prediabetes was defined as fasting plasma glucose, with values ≥100 mg/dL and <126 mg/dL. ∗Internal and external validation sets to evaluate the screening score for prediabetes (*n* = 1,551 for KNHANES 2010 and *n* = 4,566 for KNHANES 2011). ^†^Internal and external validation sets to evaluate the screening score for undiagnosed diabetes (*n* = 1,585 for KNHANES 2010 and *n* = 4,683 for KNHANES 2011).
